# Crime and physical activity measures from the SAFE and Fit Environments Study (SAFE): Psychometric properties across age groups

**DOI:** 10.1016/j.pmedr.2021.101381

**Published:** 2021-04-20

**Authors:** Scott C. Roesch, Christina M. Patch, Caterina G. Roman, Terry L. Conway, Ralph B. Taylor, Brian E. Saelens, Marc A. Adams, Kelli L. Cain, Loki Natarajan, James F. Sallis

**Affiliations:** a Department of Psychology, San Diego State University, San Diego, CA, USA; b University of California, San Diego (Department of Family Medicine and Public Health) and San Diego State University (Department of Public Health), Joint Doctoral Program in Public Health, San Diego, CA, USA; c Department of Criminal Justice, Temple University, Philadelphia, PA, USA; d Department of Family Medicine and Public Health, University of California, San Diego, CA, USA; e Department of Pediatrics and Psychiatry & Behavioral Sciences, Center for Chld Health, Behavior, and Development, University of Washington & Seattle Children’s Research Institute, Seattle, WA, USA; f College of Health Solutions, Arizona State University, Tempe, AZ, USA; g Division of Biostatistics and Bioinformatics, Department of Family Medicine and Public Health, University of California San Diego, CA, USA

**Keywords:** Crime, Physical activity, Measurement, Psychometrics, Age

## Abstract

Valid and reliable measures are needed to better understand the relationship between physical activity and crime. This paper provides a comprehensive psychometric evaluation of measures developed in the Safe and Fit Environments (SAFE) Study to assess a crime-PA conceptual framework. In addition to assessing the basic psychometric properties of each measure (e.g., variable distributions [item/scale level], internal consistency reliability), this study formally examined the measurement validity and invariance of measures across four age groups using confirmatory factor analysis. The sample (n = 2173) included 336 Adolescents (aged 12–17), 532 Young adults (aged 18–39), 838 Middle Age Adults, and 467 Older Adults (aged 66+). The psychometric evaluation of (sub)scales showed consistent factorial validity and internal consistency reliability across the majority of the measures and across the four age groups. Specifically, 14 of the 17 measures displayed statistically and practically significant factor loadings and internal consistency values in the overall sample and across the age groups. The pattern of correlations for each (sub)scale with other (sub)scales/indexes largely did not exhibit redundancy across measures. The findings expanded upon the test–retest reliability evaluation reported in Patch et al. (2019), and clarified key aspects of the construct validity of these indicators. The latter bodes well for potential utility of these indicators in future predictive models.

## Highlights

• Newly-developed measures of crime, environmental context, and activity are valid.
• Factorial validity of measures supported across four across age groups.
• Minimal measurement redundancy found across measures.

## 1 Introduction

The lack of consistent support for widely held hypotheses about crime's effect on physical activity (Carver et al., 2008; Foster, Giles-Corti, 2008) calls for an examination of the conceptual models, measures, outcomes, and analyses. The Safe and Fit Environments Study (SAFE; Patch et al., 2019) sought to evaluate the relations among crime, fear of crime, responses to crime, environmental contexts, and physical activity by developing an interdisciplinary (i.e., criminology, psychology, public health) multilevel conceptual framework with valid and reliable measures to test hypotheses derived from the framework (Patch et al., 2019). The conceptual framework is multilevel because hypothesized processes reflecting individual, social, and neighborhood/community attributes are involved in pathways linking crime and fear of crime with multiple measures of physical activity. Measures were developed for each construct of the framework, with the goal of generating items and subscales suitable for use across age groups (Adolescent [12–17], Young Adult [18–39], Middle Age Adult [40–65], Older Adult [66+]). These broader conceptual domains referenced the (a) Micro Level, representing individual factors such as cognitive assessment of crime/safety, emotional responses to crime, behavioral responses to crime/victimization/physical activity, and personal experiences/victimization; the (b) Meso Level, representing perceptions of social control and social cohesion in participants’ communities; and the (c) Macro Level, referencing crime prevention through reducing opportunities for crime in the neighborhood context. Each conceptual domain was operationalized with multiple indicators. Table 1 briefly describes the indicators.

**Table 1:** SAFE Study survey measures with conceptual definitions, response scales, number of items and sample items.

Measures	Conceptual definition, response scale, number of items, sample item
**Macrolevel: neighborhood context**	
Crime Prevention through Environmental	Reducing opportunities for crime and attractiveness of targets
Design (CPTED), 4 subscales:	Response scale: 1 = disagree strongly, 2 = disagree somewhat, 3 = agree somewhat, 4 = agree strongly
1) Surveillance	5 items; “When I walk in my neighborhood, I know there are residents or business owners watching the streets.”
2) Maintenance	3 items; “The homes, buildings, and landscaping in my neighborhood are well-maintained.”
3) Access Control	2 items; “A lot of the homes or apartment buildings in my neighborhood have fences, locked gates, entrances and/or metal security doors to keep out criminals.”
4) Territorial Reinforcement	2 items; “A lot of my neighbors have signs on their property signaling for people to keep out.”
**Mesolevel: social dynamics**	
5) Collective efficacy	Assesses informal social control, social cohesion and trust
	Response scale: 1 = disagree strongly, 2 = disagree somewhat, 3 = agree somewhat, 4 = agree strongly 9 items; “People in my neighborhood can be trusted.”
6) Neighborhood integration	Extent to which the individual knows and engages with neighbors
	Response scale: 1 = disagree strongly, 2 = disagree somewhat, 3 = agree somewhat, 4 = agree strongly 6 items; “I know many of the people in my neighborhood by name.”
**Microlevel:**	
**Individual factors, Personal factors**	
Victimization, 4 subscales:	Personal experience with victimization;
	Response scale: 0 = never, 1 = 1 time, 3.5 = 2–5 times, 6 = 6 or more times
7) Recent (≤12 months)	4 items; ”In the past 12 months, how many times have YOU been the victim of a shooting?”
8) Past (>12 month)	4 items; “PRIOR to the past 12 months, how many times have YOU ever been the victim of property crimes?”
9) Witness Crime	4 items; “In the past 12 months, how many times have you WITNESSED bullying in your neighborhood?”
10) Hearing about Crime	4 items; “In the past 12 months, how many times have you HEARD about someone in your neighborhood being attacked?”
11) Crime Information Sources	Where the respondent obtains crime information (e.g., media sources)
	Response scale: 0 = No, 1 = Yes
	10 items; “Please CIRCLE whether you get information about CRIME IN YOUR NEIGHBORHOOD from the radio.”
**Individual factors, Cognitive Assessment of Crime**	
12) Evaluation of risk	A cognitive assessment of the likelihood of crime
	Response scale: 1 = very unlikely, 2 = somewhat unlikely, 3 = somewhat likely, 4 = very likely
	12 items; “How likely is it that in the next year you will a victim of crime when you are in a local park.”)
13) Values/Incivilities	Concerns relating to the personal tolerance of the respondent to crime and incivilities
	Response scale: 1 = not present in my neighborhood, 2 = present, but not a problem, 3 = present, somewhat a problem, 4 = present, big problem
	17 items; “Extent to which the issue is a problem in your neighborhood…gang activity.”
14) Street Efficacy	Confidence in the ability to avoid crime or to find ways to be safe
	Response scale, 1 = disagree strongly, 2 = disagree somewhat, 3 = agree somewhat, 4 = agree strongly
	4 items; “I am confident I can avoid crime because I am good at “fitting in.”
**Individual factors, Emotional Responses to Crime**	
15) Fear of crime	Emotional response related to fear, worry, helplessness, and uneasy feelings
	Response scale: 1 = disagree strongly, 2 = disagree somewhat, 3 = agree somewhat, 4 = agree strongly
	13 items; “I am fearful of being a victim of crime when walking for recreation, health, or fitness in my neighborhood.”
**Individual factors, Behavioral Responses to Crime**	
16) Protective Behavior	Making victimization more challenging by minimizing the chances of being targeted
	Response scale, 1 = never, 2 = rarely, 3 = sometimes, 4 = often
	13 items; In the past 12 months when you have gone outside, how often have you taken someone with you to reduce your chances of becoming a victim of crime?”
Avoidant Behaviors, 4 Subscales	Measures taken to decrease exposure to crime
	Response scale, 1 = never, 2 = rarely, 3 = sometimes, 4 = often
17) Daylight, Alone	5 items; ”In the past 12 months, how often have you avoided going outside in your neighborhood to reduce your chance of becoming a victim of crime… when it’s daylight and you are alone?
18) Daylight, With Others	5 items; “In the past 12 months, how often have you avoided being in a local park to reduce your chance of becoming a victim of crime when it’s daylight and you are with other people?”
19) Dark, Alone	6 items; “In the past 12 months, how often have you avoided walking in places with poor lighting in your neighborhood AFTER DARK to reduce your chance of becoming a victim of crime when it’s dark and you are alone?”
20) Dark, With Others	6 items; “In the past 12 months, how often have you avoided walking for recreation, health, or fitness in your neighborhood to reduce your chance of becoming a victim of crime… when it’s dark and you are with others?”
21) Positive Avoidant Behavior	Avoidant behaviors that may result in increased physical activity
	Response scale: 0 = No, 1 = Yes
	10 items; “In the past 12 months, have you planned to be home before dark to avoid crime when you do outdoor activities?).
22) News-Related Avoidant Behavior	Changing or avoiding outdoor activities in response to crime-related news
	Response scale: 0 = No, 1 = Yes
	3 items; “I have changed or avoided outdoor activities in response to a crime-related news story that happened in my city.”
23) Obligatory Behaviors	Engaging in physical activity, regardless of crime-related perceptions, due to lack of an alternate form of transportation
	Response scale: 0 = No, 1 = Yes
	3 items; “I have to walk or bike because I don't have a car to use all the time.”
24) Community Participation	Civic engagement and involvement in neighborhood safety programs
	Response scale: 0 = No, 1 = Yes
	2 items; “In the past 12 months, have you attended a neighborhood meeting to address a neighborhood crime-related problem?”
25) No Behavioral Response	Captures the null set – i.e., participants who report they do not have any behavioral responses to crime
	Response scale: 0 = No, 1 = Yes
	2 items; “Crime affects my physical activity (walking, biking, jogging) in my neighborhood.”

The complete survey and more information item adaptation from original sources is available online at https://drjimsallis.org/measures.html.

A systematic process was used to develop and adapt survey items and scales to provide operational definitions of the constructs in the SAFE conceptual framework (Patch et al., 2019). The survey development process was guided by several principles: 1) draw from existing crime and physical activity measures when possible, 2) develop new items as needed based on focus group and expert input, 3) design items to apply across age, gender, and socio-demographic groups, and 4) minimize respondent burden by limiting length of scales and maintaining a common response format. The survey development process began with a non-systematic literature review to identify existing measures in the fields of criminology, sociology, and physical activity that related to constructs in the framework. Over the course of a year, an interdisciplinary team of experts met weekly to review existing measures and adapt them as necessary. When measures did not exist or were deemed inadequate to represent a content domain, items were generated by the team. The experts discussed the language used for items, response formats, and psychometrics (when available). Some items were adopted from existing measures without material change, but most were altered somewhat in content, format, or both.

Test-retest reliability information for these measures was provided on a subsample of participants (N = 176) by Patch et al. (2019). The majority of these measures achieved adequate reliability across age groups. Given the construction of “new” measures for the SAFE study, it is vital to conduct an integrated psychometric evaluation to confirm the construct validity of each measure before these measures can be used in predictive models. Gaining important additional information about the construct validities of these indicators was the primary purpose of the present paper. Of specific interest in the current analysis is the invariance of these measures (and by implication, constructs) across age groups. The use of large sample sizes for each age group provides an opportunity to validate the majority of the measures and establish construct validity, internal consistency reliability, and concurrent/discriminant validity across the lifespan. Only after it has been demonstrated that the constructs of interest are being measured similarly and independently in each age group can one attribute substantive differences/associations to substantive factors rather than noncomparability of the measurement instrument.

There are multiple aspects to validity, conceptually unified under construct validation (Messick, 1995). The structural aspect of validity refers to the correspondence between the scoring of the measurement instrument and the hypothesized constructs and involves examination at the level of the items (also referred to as measurement validity). The extent of evidence required for demonstrating validity depends on the types of inferences one wishes to make from the obtained scores, but at a minimum, the structure of the measure should be established. This is referred to as factorial validity, and is traditionally established using factor analysis (exploratory, confirmatory). When using measurement instruments that have been developed for different age groups, an added concern is the extent to which the measurement instrument has the same structure across age groups. At the initial level of analysis, this is referred to as configural or pattern invariance (Roesch et al., 2013, Vandenberg and Lance, 2000). In the present study, factor loadings in each age group were tested to determine if they are descriptively invariant (equivalent). If this initial invariance is observed, one can infer that a measurement instrument yields scores that can be interpreted in a similar fashion across different populations or groups, in this case different age groups.

In sum, the current study, a comprehensive psychometric evaluation of the SAFE measures was undertaken. In addition to assessing the basic psychometric properties of each measure (e.g., variable distributions, internal consistency reliability), this study formally examined the factorial validity and invariance of measures using confirmatory factor analysis (CFA). Not all measures from the SAFE study were amenable to CFA. Of the 25 measures identified in Table 1, 17 were subjected to psychometric evaluation that specifically included CFA; these measures are referred to as (sub)scales throughout the manuscript. Eight measures could not be evaluated using CFA for a number of reasons, including operationalization by only two items for a given measure, use of a binary response scale, or the lack of an underlying continuum represented by the response scale. These measures are referred to as (summative) indexes through the manuscript. Physical activity measures used in the study were generally previously-evaluated measures, and they were not further examined in present analyses.

## 2 Materials and methods

### 2.1 Participants

The survey was administered to study participants of the Safe and Fit Environments Study (SAFE Study) recruited from four metropolitan US regions: Baltimore counties Maryland/Washington DC; Seattle/King County, Washington; San Diego County, California; and Phoenix/Maricopa County, Arizona. Most participants in the SAFE Study were re-recruited from one of four previous studies conducted by the same research team: the Neighborhood Quality of Life Study (adults; Sallis et al., 2009), the Senior Neighborhood Quality of Life Study (older adults; King et al., 2011), the Teen Environment and Neighborhood Study (adolescents; Carlson et al., 2015), and the Neighborhood Impact on Kids Study (children; Frank et al., 2012, Saelens et al., 2012). Recruitment of new participants was conducted in the same regions, with oversampling from high-crime and low-socioeconomic status areas, as well as from a new region (WalkIT Arizona; Adams et al., 2019).

In the present SAFE Study, the research team sampled to achieve a balance of participants from high- and low-crime neighborhoods and across four current age groups: 2,173 participants comprised the sample, with 336 participants in the Adolescent group (12–17), 532 participants in the Young Adult group (18–39), 838 participants in the Middle Age Adult group (40–65), and 467 participants in the Older Adult group (66+). Additional demographic information is provided in Table 2.

**Table 2:** Demographics by overall sample and by age group.

Variable	Overall	Adolescents	Young Adults	Middle Age Adults	Older Adults
		(12–17)	(18–39)	(40–65)	(66+)
Number of participants	2173	336	532	838	467
Sex, % female	60.6	57.1	61.3	65.8	52.9
Household income, %					
<$20 K	8.5	6.3	12.0	5.4	11.6
$20 K-$39 K	12.3	6.0	15.0	9.8	18.2
$40 K-$59 K	14.8	8.0	16.9	14.3	18.0
$60 K-$79 K	13.1	10.4	16.2	12.2	13.1
$80 K-$99 K	11.8	12.8	11.1	12.8	10.1
$100 K-$119 K	11.9	14.3	10.0	14.0	8.6
>$120 K	24.3	35.7	16.4	30.0	15.0
Missing	3.5	6.5	2.4	1.7	5.6
Block Group Median Income, %Low	49.3	38.7	55.3	47.1	54.2
Block Group Crime Score, %Low	51.1	55.4	51.9	49.9	49.3

### 2.2 Measures

A brief description of each (sub)scale and index is provided in Table 1. The complete survey and more information about item adaptation from original sources is available online at https://drjimsallis.org/measures.html (see also 3).

### 2.3 Statistical approach

The data for each measure in Table 1 were subjected to basic psychometric analysis. For the 17 measures for which CFA was used, descriptive statistics at the item level were evaluated to identify items with low variability and non-normal distributions. Subsequent to this, CFA models were tested for each measure to establish the best-fitting, unidimensional measurement model in the overall sample and stratified by age group. Traditionally, the likelihood ratio chi-square test has been reported but sparingly used to determine whether a model fits well. This test statistic has been identified as unsatisfactory for numerous reasons, including the heavy reliance of this statistic on sample size (Hoyle, 2000). While the use of alternative descriptive fit indices and the values used to determine overall model fit is contentious (Bentler, 2007, Marsh et al., 2004), three descriptive fit indexes have been generally recommended (Hu and Bentler, 1999): (a) the Comparative Fit Index (CFI; Bentler, 1990), a relative index of model fit with values >0.90 indicating acceptable model fit; (b) the root mean square error of approximation (RMSEA; Steiger, 1990), an absolute index of overall model fit with values less than 0.08 indicative of acceptable model fit, and (c) standardized root mean-square residual (Bentler, 1990, an absolute index of overall model fit with values less than 0.08 indicative of acceptable model fit. However, given the exploratory nature of this psychometric analysis, these more liberal threshold values were used to indicate reasonably acceptable fit. All CFA models were estimated with the MPlus software version 8.1 (Muthén and Muthén, 2019) and used both Maximum Likelihood-Robust and Weighted Least Squares estimation procedures.

Finally, descriptive statistics at the (sub)scale/index level were examined for all 25 measures to identify (sub)scales/indexes with low variability items and non-normal distributions. Correlations among all measures were examined to determine if redundancy (i.e., correlations >0.70) existed. These correlations were evaluated for the overall sample and for each age group. These correlations were evaluated for the overall sample and for each age group. Given the relatively large size of the overall sample and the sample size of each age group, statistical significance of these correlations should be interpreted with caution.

## 3 Results

Descriptive statistics, confirmatory factor analyses, and correlations among measures are presented in Table 3, Table 4, Table 5, Table 6, Table 7 for the overall sample and by age group. Specifically, the tables present item-level descriptive statistics (Table 3), standardized factor loadings from the CFAs and internal consistency coefficients (Table 4), (sub)scale- and index-level descriptive statistics (Table 5), and correlations among all (sub)scales in the overall sample (Table 6) and by age group (Table 7). The results are presented below by conceptual level of assessment (Macro, Meso, Micro).

**Table 3:** Item-level descriptive statistics: Overall and by age group.

SCALE	Overall (n=2173)	12-17 (n=336)	18-39 (n=532)	40-65 (n=838)	66+ (n=467)
	M(SD)	M(SD)	M(SD)	M(SD)	M(SD)
CPTED					
Surveillance	1.29-3.06 (0.83-0.90)	1.90-2.93 (0.86-0.92)	2.22-2.99 (0.83-0.98)	2.34-3.09 (0.79-0.85)	2.39-3.17 (0.84-0.94)
Maintenance	2.21-3.38 (0.69-0.88)	3.04-3.26 (0.72-0.95)	3.03-3.22 (0.74-0.88)	3.34-3.45 (0.63-0.80)	3.30-3.50 (0.64-0.90)
**Collective Efficacy**	1.79-3.40 (0.69-0.96)	1.74-2.36 (0.68-0.96)	1.75-2.74 (0.68-0.95)	1.52-2.32 (0.65-0.92)	1.60-2.39 (0.68-0.97)
**Neighborhood Integration**	1.69-2.91 (0.90-1.17)	2.10-3.03 (0.88-1.14)	1.54-2.04 (0.86-1.12)	1.69-2.96 (0.86-1.16)	1.57-2.97 (0.86-1.17)
**Victimization**					
Recent	0.01-0.06 (0.09-0.51)*	0.01-0.24 (0.10-0.64)*	0.01-0.27 (0.10-0.66)*	0.01-0.13 (0.09-0.40)*	0.01-0.14 (0.08-0.43)*
Past	0.03-0.60 (0.20-0.91)*	0.01-0.42 (0.11-0.88)*	0.03-0.63 (0.23-1.88)*	0.04-0.84 (0.23-0.96)*	0.01-0.54 (0.13-0.79)*
Witnessing crime	0.03-0.25 (0.22-0.65)*	0.04-0.37 (0.25-0.75)*	0.04-0.40 (0.25-0.82)*	0.03-0.18 (0.20-0.56)*	0.02-0.13 (0.19-0.48)*
Hearing about crime	0.19-.081 (0.53-0.96)*	0.15-0.37 (0.49-0.94)*	0.27-0.82 (0.66-1.03)*	0.19-0.93 (0.51-0.96)*	0.12-0.66 (0.41-0.86)*
**Evaluation of Risks**	1.12-1.56 (0.42-0.72)	1.11-1.60 (0.39-0.76)	1.13-1.65 (0.49-0.91)	1.11-1.56 (0.36-0.63)	1.10-1.57 (0.33-0.70)
**Values/Incivilities**	1.14-1.76 (0.46-0.82)	1.11-2.01 (0.44-0.88)	1.21-1.88 (0.57-0.81)	1.11-1.77 (0.40-0.77)	1.11-1.70 (0.40-0.80)
**Street Efficacy**	2.50-3.00 (0.78-0.93)	2.86-3.10 (0.74-0.90)	2.57-3.06 (0.72-0.93)	2.40-3.03 (0.73-0.89)	2.34-2.95 (0.82-0.95)
**Fear of Crime**	1.26-2.12 (0.55-0.94)	1.36-2.37 (0.57-0.98)	1.31-2.27 (0.60-0.96)	1.21-2.04 (0.49-0.85)	1.24-2.05 (0.49-0.91)
**Protective Behaviors**	1.18-2.67 (0.62-1.29)	1.19-2.38 (0.63-1.29)	1.35-2.80 (0.81-1.27)	1.14-2.82 (0.55-1.30)	1.06-2.47 (0.36-1.32)
**Avoidant Behaviors**					
Daylight, Alone	1.18-1.29 (0.54-0.84)	1.28-1.40 (0.69-0.91)	1.22-1.33 (0.58-0.82)	1.10-1.23 (0.42-0.73)*	1.14-1.28 (0.51-0.75)*
Daylight, Others	1.11-1.26 (0.46-0.74)	1.17-1.34 (0.53-0.84)	1.13-1.31 (0.48-0.80)*	1.06-1.21 (0.33-0.69)*	1.13-1.23 (0.50-0.68)*
Dark, Alone	1.41-2.00 (0.84-1.18)	1.81-2.18 (1.13-1.20)	1.72-2.15 (1.04-1.18)	1.50-1.91 (0.91-1.15)	1.61-1.87 (0.97-1.16)
Dark, Others	1.36-1.66 (0.73-1.01)	1.53-1.80 (0.88-1.06)	1.44-1.76 (0.81-1.06)	1.22-1.56 (0.57-0.95)	1.35-1.61 (0.73-1.03)

Note. All values reported are ranges of mean values (standard deviations) across the individual items.

*Statistical non-normality indicated when skewness, kurtosis values > |2| and SD > Mean

**Table 4:** Standardized factor loadings and internal consistency values: Overall and by age group.

SCALE	Overall (n=2173)		12-17 (n=336)		18-39 (n=532)		40-65 (n=838)		66+ (n=467)	
	FL	α	FL	α	FL	α	FL	α	FL	α
CPTED										
Surveillance	0.22-0.91	0.59	0.19-0.92	0.61	0.09-0.95	0.55	0.24-0.94	0.57	0.16-0.83	0.62
Maintenance	0.62-0.86	0.68	0.66-0.81	0.68	0.65-0.86	0.74	0.52-0.91	0.62	0.59-0.84	0.64
**Collective Efficacy**	0.51-0.85	0.88	0.55-0.86	0.86	0.50-0.84	0.88	0.51-0.85	0.87	0.47-0.85	0.87
**Neighborhood Integration**	0.70-0.92	0.87	0.70-0.88	0.84	0.80-0.93	0.89	0.73-0.92	0.88	0.51-0.90	0.82
**Victimization**										
Recent	0.54-1.00	0.38	0.08-1.00	0.46	0.30-0.95	0.35	0.64-1.00	0.35	0.40-1.00	0.45
Past	0.63-0.89	0.57	0.64-0.96	0.48	0.59-0.87	0.55	0.71-0.90	0.62	0.44-1.00	0.48
Witnessing crime	0.76-0.99	0.68	0.74-0.90	0.66	0.81-0.98	0.72	0.67-10.00	0.56	0.83-0.98	0.70
Hearing about crime	0.71-0.94	0.74	0.60-0.95	0.66	0.75-0.96	0.81	0.73-0.92	0.72	0.67-0.93	0.65
**Evaluation of Risks**	0.74-0.96	0.92	0.72-0.95	0.91	0.78-0.97	0.94	0.71-0.96	0.92	0.75-0.96	0.91
**Values/Incivilities**	0.63-0.89	0.91	0.56-0.88	0.88	0.58-0.90	0.92	0.54-0.89	0.91	0.58-0.91	0.91
**Street Efficacy**	0.73-0.89	0.82	0.67-0.91	0.81	0.72-0.88	0.81	0.71-0.90	0.81	0.74-0.90	0.84
**Fear of Crime**	0.63-0.92	0.92	0.58-0.93	0.92	0.56-0.94	0.92	0.65-0.94	0.92	0.66-0.93	0.92
**Protective Behaviors**	0.32-0.84	0.90	0.31-0.87	0.90	0.31-0.87	0.89	0.25-0.84	0.87	0.26-0.84	0.84
**Avoidant Behaviors**										
Daylight, Alone	0.86-0.95	0.87	0.82-0.95	0.88	0.86-0.96	0.89	0.88-0.96	0.86	0.82-0.98	0.86
Daylight, Others	0.88-0.95	0.85	0.80-0.96	0.86	0.83-0.96	0.86	0.87-0.96	0.81	0.86-0.97	0.86
Dark, Alone	0.88-0.96	0.93	0.87-0.94	0.93	0.87-0.96	0.93	0.87-0.96	0.93	0.85-0.96	0.93
Dark, Others	0.86-0.96	0.92	0.83-0.95	0.92	0.88-0.96	0.93	0.86-0.95	0.91	0.85-0.97	0.92

Note. All FL values reported are ranges across the individual items. Descriptive fit indices (comparative fit index [CFI], standardized root mean square residual [SRMR] were indicative of well-fitting models.

FL = standardized factor loadings; α = Cronbach’s alpha

**Table 5:** Descriptive statistics for all (sub)scale scores and summative indexes: Overall and by age group.

SCALE	Overall (n=2173) M(SD)	12-17 (n=336) M(SD)	18-39 (n=532) M(SD)	40-65 (n=838) M(SD)	66+ (n=467) M(SD)
CPTED					
1) Surveillance	2.64 (0.47)	2.49 (0.49)	2.58 (0.46)	2.73 (0.42)	2.66 (0.52)
2). Maintenance	3.30 (0.60)	3.14 (0.65)	3.15 (0.64)	3.41 (0.53)	3.40 (0.59)
3). Access Control	1.94 (0.76)	2.04 (0.73)	2.02 (0.73)	1.87 (0.76)	1.91 (0.81)
4). Territorial Reinforcement	1.77 (0.67)	1.88 (0.65)	1.79 (0.67)	1.74 (0.65)	1.74 (0.71)
5) Collective Efficacy	2.68 (0.46)	2.79 (0.43)	2.53 (0.49)	2.71 (0.42)	2.71 (0.48)
6) Neighborhood Integration	2.30 (0.78)	2.45 (0.76)	2.02 (0.79)	2.37 (0.79)	2.39 (0.70)
**Victimization**					
7). Recent	0.08 (0.21)*	0.11 (0.24)*	0.12 (0.25)*	0.06 (0.17)*	0.06 (0.19)*
8) Past	0.32 (0.45)	0.21 (0.36)*	0.34 (0.47)	0.42 (0.50)	0.20 (0.40)*
9) Witnessing crime	0.13 (0.33)*	0.18 (0.36)*	0.20 (0.43)*	0.10 (0.26)*	0.08 (0.27)*
10) Hearing about crime	0.40 (0.56)	0.38 (0.51)	0.50 (0.69)	0.41 (0.53)	0.29 (0.43)
11) Crime Information Sources	4.59 (2.21)	3.80 (2.33)	4.25 (2.31)	4.53 (2.10)	4.25 (2.14)
12) Evaluation of Risks	1.27 (0.40)	1.29 (0.39)	1.34 (0.46)	1.24 (0.35)	1.25 (0.38)
13) Values/Incivilities	1.37 (0.40)	1.40 (0.38)	1.45 (0.46)	1.34 (0.37)	1.31 (0.39)
14) Street Efficacy	2.80 (0.68)	2.98 (0.63)	2.81 (0.66)	2.77 (0.64)	2.73 (0.73)
15) Fear of Crime	1.57 (0.53)	1.68 (0.57)	1.66 (0.56)	1.50 (0.48)	1.52 (0.52)
16) Protective Behaviors	1.73 (0.64)	1.75 (0.67)	1.86 (0.69)	1.71 (0.61)	1.59 (0.55)
**Avoidant Behaviors**					
17). Daylight, Alone	1.21 (0.51)	1.33 (0.61)	1.24 (0.54)	1.15 (0.45)	1.20 (0.50)
18) Daylight, Others	1.15 (0.43)	1.21 (0.50)	1.18 (0.46)	1.11 (0.34)	1.17 (0.46)
19) Dark, Alone	1.78 (0.94)	2.02 (1.01)	1.87 (0.97)	1.66 (0.88)	1.73 (0.93)
20) Dark, Others	1.47 (0.75)	1.62 (0.82)	1.55 (0.81)	1.35 (0.65)	1.48 (0.78)
21) Positive Avoidant Behaviors	1.61 (2.20)	1.81 (2.21)	1.88 (2.39)	1.34 (2.05)	1.62 (2.18)
22) News-Related Avoidant Behaviors	0.33 (0.71)*	0.44 (0.79)	0.46 (0.82)	0.24 (0.61)*	0.27 (0.66)*
23) Obligatory Behaviors	0.48 (0.93)	1.10 (1.08)	0.61 (1.04)	0.22 (0.69)*	0.21 (0.68)*
24) Community Participation	0.13 (0.42)*	0.12 (0.35)*	0.07 (0.29)*	0.14 (0.44)*	0.21 (0.52)*
25) No Behavioral Response	1.33 (0.65)	1.31 (0.63)	1.41 (0.69)	1.28 (0.61)	1.33 (0.70)

*Statistical non-normality (skewness, kurtosis > |2| and SD > Mean)

**Table 6:** Correlations among study measures: Overall.

SCALE	2.	3.	4.	5.	6.	7.	8.	9.	10.	11.	12.	13.	14.	15.
1. CPTED: Surveillance	0.32*	0.10*	0.08*	0.32*	0.27*	-0.05*	0.01	-0.01	0.05*	0.22*	-0.04	0.02	0.11*	-0.08*
2. CPTED: Maintenance		-0.09*	-0.25*	0.36*	0.19*	-0.23*	-0.11*	-0.24*	-0.20*	0.02	-0.40*	-0.448	0.07*	-0.39*
3. CPTED: Access Control			0.36*	-0.09*	-0.08*	0.10*	-0.01	0.11*	0.06*	0.11*	0.24*	0.16*	0.11*	0.25*
4. CPTED: Territorial Reinforcement				-0.05*	0.01	0.18*	0.09*	0.20*	0.21*	0.18*	0.36*	0.36*	0.04	0.35*
5. Collective Efficacy					0.57*	-0.16*	-0.14*	-0.17*	-0.15*	12*	-0.22*	-0.27*	0.15*	-0.20*
6. Neighborhood Integration						-0.08*	-0.07*	-0.04	-0.01	0.21*	-0.12*	-0.09*	0.09*	-0.12*
7. Victimization: Recent							0.40*	0.53*	0.38	0.04	0.36*	0.42*	-0.02	0.28*
8. Victimization: Past								0.36*	0.38*	0.04	0.17*	0.31*	-0.08*	0.09*
9. Victimization: Witnessing crime									0.55*	0.11*	0.35*	0.48*	0.04	0.26*
10. Victimization: Hearing about crime										0.18*	0.35*	0.54*	-0.04	0.26*
11. Crime Information Sources											0.14*	0.15*	0.09*	0.14*
12. Evaluation of Risks												0.57*	-0.10*	0.65*
13. Values/Incivilities													-0.03	0.50*
14. Street Efficacy														-0.08*
15. Fear of Crime														
16. Protective Behaviors														
17. Avoidant Behav.: Daylight, Alone														
18. Avoidant Behav.: Daylight, Others														
19. Avoidant Behav.: Dark, Alone														
20. Avoidant Behav.: Dark, Others														
21. Positive Avoidant Behaviors														
22. News-Related Avoidant Behaviors														
23. Obligatory Behaviors														
24. Community Participation														
25. No Behavioral Response														

**SCALE**	**16.**	**17.**	**18.**	**19.**	**20.**	**21.**	**22.**	**23.**	**24.**	**25.**
1. CPTED: Surveillance	0.05*	-0.07*	-0.05*	-0.03	-0.04	0.00	-0.03	-0.02	0.11*	-0.02
2. CPTED: Maintenance	-0.23*	-0.25*	-0.22*	-0.25*	-0.28*	-0.28*	-0.19*	-0.10*	-0.04	-0.32*
3. CPTED: Access Control	0.18*	0.20*	0.21*	0.16*	0.18*	0.17*	0.10*	0.12	0.04	0.21*
4. CPTED: Territorial Reinforcement	0.30*	0.27*	0.26*	0.26*	0.29*	0.29*	0.20*	0.14*	0.11*	0.32*
5. Collective Efficacy	-0.17*	-0.16*	-0.14*	-0.16*	-0.18*	-0.15*	-0.12*	-0.07*	0.04	–23*
6. Neighborhood Integration	-0.07*	-0.08*	-0.09*	-0.05*	0.08*	-0.06*	-0.04	-0.08	0.12*	-0.12*
7. Victimization: Recent	0.27*	0.20*	0.19*	0.20*	0.21*	0.21*	0.21*	0.15*	0.10*	0.28*
8. Victimization: Past	0.18*	0.06*	0.03	0.08*	0.05*	0.11*	*0.10	0.01	0.07*	0.18*
9. Victimization: Witnessing crime	0.29*	0.24*	0.22*	0.23*	0.25*	0.27*	0.23*	0.17*	0.14*	0.28*
10. Victimization: Hearing about crime	0.29*	0.19*	0.14*	0.28*	0.25*	0.29*	0.23*	0.07*	0.19*	0.31*
11. Crime Information Sources	0.20*	0.10*	0.12*	0.11*	0.12*	0.14*	0.16*	-0.01	0.18*	0.13*
12. Evaluation of Risks	0.44*	0.45*	0.44*	0.48*	0.50*	0.48*	0.36*	0.14*	0.12*	0.58*
13. Values/Incivilities	0.41*	0.35*	0.30*	0.42*	0.41*	0.44*	0.33*	0.15*	0.18*	0.53*
14. Street Efficacy	0.01	-0.04	-0.02	-0.12*	-0.07*	-0.07*	-0.05*	0.11*	0.02	-0.07*
15. Fear of Crime	0.51*	0.53*	0.46*	0.55*	0.57*	0.53	0.41*	0.12*	0.08*	0.57*
16. Protective Behaviors		0.46*	0.40*	0.61*	0.56*	0.64*	0.42*	0.12*	0.11*	0.48*
17. Avoidant Behav.: Daylight, Alone			0.87*	0.60*	0.70*	0.52*	0.41*	0.09*	0.09	0.53*
18. Avoidant Behav.: Daylight, Others				0.47*	0.65*	0.46*	0.36*	0.11*	0.09*	0.49*
19. Avoidant Behav.: Dark, Alone					0.85*	0.67*	0.43*	0.10*	0.10*	0.52*
20. Avoidant Behav.: Dark, Others						0.65*	0.45*	0.09*	0.10*	0.57*
21. Positive Avoidant Behaviors							0.45*	0.13*	0.15*	0.57*
22. News-Related Avoidant Behaviors								0.12*	0.07*	0.41*
23. Obligatory Behaviors									-0.05	0.12*
24. Community Participation										0.17*
25. No Behavioral Response										

*p < .01

**Table 7:** Correlations among study measures by age group: 12–17, 18–39, 40–65, 66+.

SCALE	2	3	4	5	6	7	8	9	10	11	12	13	14	15
1. CPTED: Surveillance	0.57*	0.13*	0.04	0.27*	0.26*	−0.04	0	0.03	0.06	0.18*	−0.06	0.02	0.07	−0.07
	0.28*	0.06	0.16*	0.31*	0.23*	−0.02	−0.03	0.01	−0.02	0.17*	−0.03	0	0.08	−0.07
	0.25*	0.09*	0.03	0.37*	0.32*	−0.06	−0.01	−0.04	0.07	0.22*	-0.08*	0.02	0.11*	-0.14*
	0.32*	0.22*	0.15*	0.31*	0.25*	0	0	0.08	0.15*	0.26*	0.10*	0.11*	0.23*	-0.09*
2. CPTED: Maintenance		0.02	-0.20*	0.46*	0.33*	-0.21*	-0.16*	-0.16*	-0.15*	0.07	-0.43*	-0.42*	0.18*	-0.36*
		−0.07	-0.32*	0.41*	0.17*	-0.22*	−13*	-0.31*	-0.31*	−0.07	-0.44*	-0.53*	0.04	-0.48*
		-0.13*	-0.27*	0.29*	0.11*	-0.23*	-0.13*	-0.23*	-0.18*	0	-0.40*	-0.41*	0.07	-0.38*
		−0.06	-0.16*	0.33*	0.18*	-0.15*	-0.12*	-0.10*	−0.04	0.04	-0.27*	-0.29*	0.12*	-0.23*
3. CPTED: Access Control			0.30*	0.05	0.01	0.07	0	0.07	0.07	0.05	0.12*	0.11	0.1	0.13*
			0.36*	−0.06	−0.05	0.08	0	0.06	0.08	0.13*	0.22*	0.09	0.06	0.22*
			0.36*	-0.09*	-0.11*	0.09*	−0.01	0.11*	0.05	0.07	0.24*	0.17*	0.07	0.25*
			0.40*	−0.05	−0.09	0.12*	0.01	0.16*	0.05	0.24*	0.31*	0.23*	0.19*	0.31*
4. CPTED: Territorial Reinforcement				0.05	−0.02	0.17*	0.13*	0.14*	0.13*	0.09	0.34*	0.36*	0.1	0.32*
				−0.06	0.02	0.21*	0.09*	0.24*	0.27*	0.25*	0.38*	0.37*	0.03	0.42*
				−0.05	0.02	0.16*	0.10*	0.20*	0.22*	0.16*	0.35*	0.35*	−0.02	0.33*
				-0.12*	−0.03	0.17*	0.10*	0.20*	0.17*	0.25*	0.38*	0.38*	0.07	0.32*
5. Collective Efficacy					0.54*	-0.23*	-0.27*	-0.20*	-0.16*	0.1	-0.26*	-0.24*	0.14*	-0.17*
					0.55*	-0.13*	-0.13*	-0.21*	-0.22*	0.05	-0.23*	-0.31*	0.11	-0.25*
					0.57*	-0.09*	-0.11*	-0.13*	−0.07	0.17*	-0.22*	-0.22*	0.14*	-0.20*
					0.54*	-0.14*	-0.10*	-0.09*	-0.09*	0.20*	-0.14*	-0.25*	0.20*	-0.15*
6. Neighborhood Integration						-0.16*	-0.15*	−0.08	−0.04	0.20*	-0.19*	-0.13*	0.11	−0.11
						−0.05	−0.05	0.04	−0.02	0.15*	−0.06	−09*	0.04	−0.08
						−0.04	−0.03	−0.03	−0.05	0.25*	-0.11*	−0.01	0.05	-0.14*
						−0.05	−0.07	−0.05	0.05	0.29*	-0.10*	-0.10*	0.21*	−0.09
7. Victimization: Recent							0.65*	0.48*	0.44*	−0.02	0.45*	0.40*	−0.08	0.24*
							0.43*	0.60*	0.41*	0.13*	0.33*	0.41*	0.05	0.28*
							0.32*	0.37*	0.31*	0.02	0.30*	0.39*	−0.05	0.23*
							0.48*	0.64*	0.37*	0.07	0.37*	0.45*	−0.05	0.36*
8. Victimization: Past								0.55*	0.58*	0.02	0.39*	0.37*	-0.14*	0.18*
								0.34*	0.36*	0.06	0.24*	0.32*	−0.03	0.26*
								0.31*	0.34*	−0.02	0.08*	0.32*	−0.07	0.06
								0.48*	0.64*	0.06	0.15*	0.31*	-0.10*	0.08
9. Victimization: Witnessing crime									0.61*	0.06	0.32*	0.36*	−0.04	0.15*
									0.61*	0.22*	0.41*	0.55*	0.12*	0.30*
									0.47*	0.07	0.30*	0.45*	−0.02	0.25*
									0.53*	0.10*	0.32*	0.46*	0	0.27*
10. Victimization: Hearing about crime										0.12	0.29*	0.41*	-0.14*	0.20*
										0.22*	0.48*	0.60*	0.02	0.35*
										0.16*	0.28*	0.54*	−0.05	0.22*
										0.20*	0.26*	0.51*	−0.08	0.19*
11. Crime Information Sources											0.03	0.14*	−0.06	0.11
											0.20*	0.19*	0.12*	0.21*
											0.10*	0.12*	0.08*	0.11*
											0.23*	0.19*	0.24*	0.19*
12. Evaluation of Risks												0.65*	-0.14*	0.64*
												0.62*	−0.05	0.65*
												0.47*	-0.14*	0.64*
												0.58*	-0.09*	0.64*
13. Values/Incivilities													−0.07	0.48*
													−0.01	0.53*
													−0.05	0.46*
													−0.07	0.49*
14. Street Efficacy														-0.13*
														-0.12*
														-0.09*
														−0.03
15. Fear of Crime														
16. Protective Behaviors														
**SCALE**	**2**	**3**	**4**	**5**	**6**	**7**	**8**	**9**	**10**	**11**	**12**	**13**	**14**	**15**
25. No Behavioral Response														

SCALE	16	17	18	19	20	21	22	23	24	25
1. CPTED: Surveillance	0	−0.05	−0.05	0.03	−0.03	0.01	0.05	0.04	0.05	−0.02
	0.06	0	−0.01	−0.02	−0.01	−0.01	0	0.05	0.05	0.02
	0	-0.12*	-0.12*	−0.05	-0.10*	−0.01	−0.04	0	0.10*	−0.05
	0.21*	−0.01	0.04	0.07	-0.11*	0.11*	0.01	0.09	0.16*	0.02
2. CPTED: Maintenance	-0.24*	-0.20*	-0.20*	-0.21*	-0.21*	-0.24*	-0.12*	0.04	−0.02	-0.30*
	-0.30*	−25*	-0.25*	-0.31*	-0.31*	-0.31*	-0.26*	-0.13*	−0.08	-0.39*
	-0.17*	-0.26*	-0.24*	-0.20*	-0.29*	-0.26*	-0.11*	−0.05	-0.11*	-0.32*
	-0.16*	-0.21*	-0.15*	-0.17*	-0.19*	-0.24*	-0.16*	0	0.02	-0.24*
3. CPTED: Access Control	0.1	0.17*	0.23*	0.02	0.09	0.03	0.01	0.02	0.11	0.12
	0.19*	0.17*	0.14*	0.17*	0.16*	0.21*	0.1	0.06	0.12	0.22*
	0.14*	0.16*	0.17*	0.14*	0.16*	0.16*	0.07	0.14*	0.02	0.23*
	0.29*	0.29*	0.28*	0.24*	0.26*	0.20*	0.17*	0.14*	0.04	0.21*
4. CPTED: Territorial Reinforcement	0.21*	0.19*	0.19*	0.20*	0.23*	0.20*	0.13*	0.09	0.17*	0.24*
	0.35*	0.32*	0.30*	0.30*	0.32*	0.36*	0.23*	0.08	0.16*	0.41*
	0.26*	0.23*	0.21*	0.22*	0.25*	0.24*	0.18*	0.19*	0.07*	0.31*
	0.38*	0.30*	0.32*	0.29*	0.34*	0.33*	0.20*	0.14*	0.12*	0.30*
5. Collective Efficacy	-0.19*	-0.20*	-0.21*	-0.13*	-0.17*	-0.15*	−0.05	−0.04	−0.04	-0.24*
	-0.22*	-0.16*	-0.13*	-0.21*	-0.21*	-0.21*	-0.19*	−0.08	−0.06	-0.27*
	-0.10*	-0.18*	-0.17*	-0.12*	-0.16*	-0.09*	−0.06	-0.17*	0	-0.19*
	-0.13*	-0.15*	-0.09*	-0.18*	-0.16*	-0.13*	-0.11*	-0.09*	0.14*	-0.18*
6. Neighborhood Integration	−0.05	−0.07	-0.12*	−0.01	−0.1	−0.05	−0.03	−0.06	0.06	-0.12*
	−0.06	−0.05	−0.04	−0.08	-0.09*	−0.07	−0.06	−0.07	0.06	-0.11*
	−0.05	-0.09*	-0.10*	−0.03	−0.07	−0.04	−0.02	-0.15*	0.12*	−0.07
	−0.03	-0.11*	−0.09	−0.06	−0.07	−0.05	−4	-0.13*	0.17*	-0.15*
7. Victimization: Recent	0.23*	0.15*	0.15*	0.17*	0.19*	0.11	0.21*	0.19*	0.07	0.31*
	0.34*	0.14*	0.13*	0.18*	0.18*	0.23*	0.21*	0.07	0.07	0.25*
	0.19*	0.26*	0.23*	0.21*	0.25*	0.25*	0.12*	0.12*	0.14*	0.33*
	0.25*	0.22*	0.23*	0.19*	0.19*	0.19*	0.26*	0.13*	0.18*	0.23*
8. Victimization: Past	0.21*	0.18*	0.19*	0.21*	0.22*	0.15*	0.25*	0.16*	0.13*	0.33*
	0.25*	0.06	0.02	0.11*	0.06	0.10*	0.13*	0.01	0.09*	0.17*
	0.14*	0.08*	0.03	0.09*	0.07	0.15*	0.06	0.04	0.05	0.19*
	0.11*	0.03	0.02	0.03	0	0.10*	0.10*	0	0.25*	0.23*
9. Victimization: Witnessing crime	0.21*	0.20*	0.24*	0.20*	0.23*	0.17*	0.22*	0.17*	0.13*	0.20*
	0.36*	0.24*	0.22*	0.21*	0.25*	0.26*	0.23*	0.08	0.13*	0.31*
	0.24*	0.29*	0.22*	0.26*	0.27*	0.34*	0.13*	0.21*	0.32*	0.15*
	0.23*	0.17*	0.17*	0.20*	0.19*	0.24*	0.29*	0.14*	0.25*	0.23*
10. Victimization: Hearing about crime	0.25*	0.20*	0.17*	0.28*	0.25*	0.24*	0.28*	0.15*	0.15*	0.31*
	0.37*	0.24*	0.19*	0.31*	0.29*	0.33*	0.29*	0.06	0.19*	0.38*
	0.19*	0.17*	0.10*	0.28*	0.24*	0.29*	0.15*	0.03	0.23*	0.29*
	0.27*	0.14*	0.12*	0.24*	0.22*	0.27*	0.19*	0	0.22*	0.22*
11. Crime Information Sources	0.16*	0.08	0.07	0.11	0.09	0.13*	0.23*	0.15*	0.1	0.1
	0.24*	0.19*	0.21*	0.19*	0.23*	0.19*	0.20*	−0.01	0.21*	0.23*
	0.16*	0.08*	0.07*	0.09*	0.09*	0.23*	0.11*	−0.02	0.16*	0.07
	0.28*	0.12*	0.16*	0.10*	0.14*	0.18*	0.17*	−0.02	0.26*	0.13*
12. Evaluation of Risks	0.47*	0.44*	0.43*	0.42*	0.45*	0.41*	0.35*	0.03	0.11	0.64*
	0.49*	0.42*	0.35*	0.56*	0.53*	0.54*	0.42*	0.13*	0.17*	0.61*
	0.38*	0.45*	0.45*	0.44*	0.48*	0.42*	0.31*	0.23*	0.14*	0.54*
	0.43*	0.52*	0.56*	0.48*	0.53*	0.49*	0.32*	0.18*	0.13*	0.54*
13. Values/Incivilities	0.41*	0.35*	0.32*	0.43*	0.42*	0.43*	0.33*	0.13*	0.18*	0.58*
	0.46*	0.35*	0.30*	0.43*	0.42*	0.44*	0.37*	0.10*	0.15*	0.56*
	0.36*	0.32*	0.28*	0.32*	0.39*	0.43*	0.26*	0.20*	0.25*	0.53*
	0.36*	0.38*	0.33*	0.43*	0.41*	0.44*	0.34*	0.14*	0.19*	0.48*
14. Street Efficacy	−0.12	−0.11	−0.03	-0.15*	-0.15*	-0.15*	-0.15*	0.06	−0.07	−13*
	−0.05	−0.05	−0.02	-0.14*	−0.07	−0.05	−0.04	0.16*	−0.01	−0.05
	−0.02	−0.03	−0.04	-0.15*	-0.12*	-0.10*	-0.17*	0.01	0.03	−0.07
	−0.09	−0.06	−0.01	-0.09*	−0.05	−0.03	0.02	−0.02	0.10*	−0.06
15. Fear of Crime	0.53*	0.50*	0.38*	0.52*	0.53*	0.44*	0.31*	0	0.17*	0.60*
	0.57*	0.54*	0.48*	0.59*	0.59*	0.59*	0.48*	0.05	0.11*	0.61*
	0.50*	0.54*	0.48*	0.52*	0.56*	0.53*	0.39*	0.13*	0.25*	0.55*
	0.40*	0.52*	0.48*	0.52*	0.55*	0.51*	0.35*	0.19*	0.13*	0.55*
16. Protective Behaviors		0.57*	0.45*	0.68*	0.65*	0.68*	0.44*	0.01	0.24*	0.55*
		0.51*	0.45*	0.66*	0.62*	0.68*	0.51*	0.15*	0.12*	0.58*
		0.41*	0.35*	0.62*	0.53*	0.64*	0.56*	0.11*	0.10*	0.42*
		0.36*	0.38*	0.49*	0.50*	0.57*	0.31*	0.11*	0.14*	0.40*
17. Avoidant Behav.: Daylight, Alone			0.82*	0.64*	0.74*	0.50*	0.32*	−0.02	0.16*	0.54*
			0.91*	0.60*	0.71*	0.54*	0.51*	0.07	0.07	0.56*
			0.88*	0.56*	0.69*	0.53*	0.41*	0.09*	0.07	0.50*
			0.87*	0.58*	0.65*	0.49*	0.34*	0.09*	0.14*	0.57*
18. Avoidant Behav.: Daylight, Others				0.41*	0.64*	0.36*	0.23*	0.06	0.14*	0.47*
				0.49*	0.65*	0.49*	0.45*	0.08	0.07	0.49*
				0.48*	0.66*	0.45*	0.35*	0.08*	0.07	0.46*
				0.46*	0.65*	0.48*	0.33*	0.16*	0.12*	0.53*
19. Avoidant Behav.: Dark, Alone					0.87*	0.65*	0.45*	0.06	0.14*	0.48*
					0.87*	0.73*	0.48*	0.02	0.09*	0.61*
					0.83*	0.70*	0.41*	0.07	0.07	0.46*
					0.84*	0.64*	0.35*	0.07	0.10*	0.47*
20. Avoidant Behav.: Dark, Others						0.36*	0.42*	0.02	0.16*	0.54*
						0.72*	0.51*	0.04	0.09*	0.64*
						0.65*	0.40*	0.03	0.12*	0.56*
						0.61*	0.36*	0.13*	0.10*	0.55*
21. Positive Avoidant Behaviors							0.44*	0.05	0.21*	0.51*
							0.51*	0.10*	0.15*	0.64*
							0.43*	0.17*	0.12*	0.54*
							0.38*	0.18*	0.20*	0.55*
22. News-Related Avoidant Behaviors								0.01	0.13*	0.39*
								0.12*	0.11*	0.49*
								0.12*	0.03	0.39*
								0.10*	0.14*	0.33*
23. Obligatory Behaviors									0.02	0.07
									−0.02	0.11*
									−0.04	0.15*
									−0.02	0.19*
24. Community Participation										0.26*
										0.21*
										0.15*
										0.15*

Note. Age group by cell: 12–17, 18–39, 40–65, 66+, *p < .01

### 3.1 Macro-level: neighborhood context

Means and standard deviations for the Crime Prevention through Environmental Design (CPTED) measures all indicated reasonable normality and sufficient variation at the item level and across age groups (Table 3). The configural invariance model fit reasonably well for CPTED Surveillance (RMSEA = 0.114 to 0.156, SRMR = 0.057 to 0.078, CFI = 0.900 to 0.970). However, the standardized factor loadings for two of the items were not practically significant (loadings <0.25) in the overall sample and for each age group (“There are many places in my neighborhood where criminals could wait for victims without being seen” and “The police patrol my neighborhood frequently”).

Moreover, internal consistency values were relatively low (see Table 4). Conversely, all items for CPTED-Maintenance had large loadings, and internal consistency values were reasonable. At the subscale level, the descriptive statistics and normality for the four CPTED subscales exhibited similar means and standard deviations across age groups (see Table 5). No excessive non-normality was exhibited. Correlations with other study measures did not exhibit redundancy with other measures and correlation patterns were similar across the four age groups (see Table 6, Table 7).

### 3.2 Meso-level: social dynamics

Means and standard deviations for the Collective Efficacy and Neighborhood Integration also indicated reasonable normality and sufficient variation at the item level. The configural invariance model fit reasonably well for both Collective Efficacy (RMSEA = 0.119 to 0.184, SRMR 0.067 to 0.081, CFI = 0.906 to 0.925) and Neighborhood Integration (RMSEA = 0.156 to 0.214, SRMR 0.042 to 0.067, CFI = 0.935 to 0.981). The standardized factor loadings for both measures were all large, significant, and similar across age groups. The internal consistency values were all large. At the scale level, the means and standard deviations were similar across age groups and non-normality was not evident. While the two measures within this domain did exhibit a strong correlation with each other (*r*s ranged from 0.54 to 0.57), correlations with other study measures did not exhibit redundancy with other measures, and correlation patterns were similar across the four age groups.

### 3.3 Micro-level: individual factors (personal experiences)

Means and standard deviations at the item level for the four Victimization subscales all indicated non-normality with significant positive skew. There was infrequent endorsement of victimization. The configural invariance model fit reasonably well for all four Victimization subscales: Recent (RMSEA = 0.001 to 0.069, SRMR = 0.009 to 0.159, CFI = 0.953 to 1), Ever (RMSEA = 0.008 to 0.110, SRMR = 0.015 to 0.073, CFI = 0.957 to 1), Witnessing Crime (RMSEA = 0.001 to 0.072, SRMR = 0.019 to 0.078, CFI = 0.971 to 1), Hearing about Crime (RMSEA = <0.001 to 0.001, SRMR = 0.001 to 0.027, CFI = 0.999 to 1). The overwhelming majority of factor loadings were large, significant, and similar across age groups. The exception was a single item in the 12–17 age group for the Recent subscale (item: In the past 12 months, how many times have you been the victim of property crimes [including theft, motor vehicle theft, burglary, vandalism]). This item did not significantly load (standardized value = 0.08). Internal consistency values were relatively low for the Recent and Past subscales but were consistently reasonable for the Witnessing Crime and Hearing about Crime subscales. At the subscale level, these measures continued to exhibit severe non-normality with significant positive skew.

The Crime Information Resources summated index exhibited an approximately normal distribution with significant variation. All measures within this domain exhibited similar means and standard deviations across age groups. Some correlations among the four Victimization subscales were strong, but none exceeded 0.65. Correlations with Crime Information Resources and other measures did not indicate redundancy.

### 3.4 Micro-level: individual factors (Cognitive Assessment of Crime)

Item-level means and standard deviations for Evaluation of Risks and Values/Incivilities indicated relatively low endorsement of some items; this did not, however, result in significant non-normality. The Street Efficacy measure approximated normality well. There was significant variation for all three measures. The configural invariance model fit reasonably well for Evaluation of Risks (RMSEA = 0.083 to 0.094, SRMR 0.045 to 0.056, CFI = 0.968 to 0.986), Values/Incivilities, (RMSEA = 0.040 to 0.060, SRMR 0.044 to 0.064, CFI = 0.962 to 0.977), and Street Efficacy (RMSEA = 0.041 to 0.141, SRMR 0.009 to 0.025, CFI = 0.988 to 0.999). The standardized factor loadings for all three measures were large, significant, and similar across age groups. The internal consistency values were also large. Similar to what was observed at the item-level, at the scale level, the means and standard deviations were similar across age groups, and non-normality was not evident. Significant and strong correlations were found between Evaluation of Risks and Values/Incivilities (*r*s ranged from 0.47 to 0.65). Strong, positive relationships were also found between these two measures and No Behavioral Response (*r*s ranged from 0.48 to 0.64), but not strong enough to indicate redundancy. Correlations with other study measures did not exhibit redundancy with other measures, and correlation patterns were similar across the four age groups. No strong correlations were found for the Street Efficacy measure with other study measures, and the pattern of correlations was similar across age groups.

### 3.5 Micro-level: individual factors (Emotional responses to Crime)

Means and standard deviations for the Fear of Crime measure indicated reasonable normality and sufficient variation at the item level. The configural invariance model fit reasonably well (RMSEA = 0.115 to 0.124, SRMR 0.056 to 0.070, CFI = 0.948 to 0.966). The standardized factor loadings for both measures were all large, significant, and similar across age groups. The internal consistency values were all large. At the scale level, the means and standard deviations were similar across age groups, and only mild non-normality was evident. This measure did exhibit a strong correlation with the Values/Incivilities measure (*r* = 0.65), but correlations with other study measures did not exhibit redundancy.

### 3.6 Micro-level: individual factors (Behavioral responses to Crime)

Means and standard deviations for the Protective Behaviors measure indicated reasonable normality and sufficient variation at the item level. The configural invariance model fit reasonably well (RMSEA = 0.068 to 0.078, SRMR 0.050 to 0.080, CFI = 0.962 to 0.980). The standardized factor loadings for this measure were generally large, all were significant, and they were similar across age groups. The internal consistency values were all large. At the scale level, the means and standard deviations were similar across age groups but mild non-normality was evident across age groups. This measure exhibited strong correlations with Avoidant Behaviors (Dark, Alone subscale and the Positive Avoidant Behaviors measure, respectively [*r*s ranged from 0.61 to 0.68]), but correlations with other study measures did not indicate redundancy.

Means and standard deviations at the item level for the four Avoidant Behaviors subscales all indicated some non-normality with positive skew. There was generally a low endorsement for the items from these subscales. In general, mild non-normality was evident for the majority of items. There was, however, significant non-normality (positive skew) for both the Daylight, Alone and Daylight, Others subscales in both the 40–65 and 66+ age groups; also for the Daylight, Others subscale in the 18–39 age group. The configural invariance model fit reasonably well for all four subscales: Daylight, Alone (RMSEA = 0.028 to 0.070, SRMR = 0.012 to 0.018, CFI = 0.996 to 1), Daylight, Others (RMSEA = 0.001 to 0.060, SRMR = 0.009 to 0.018, CFI = 0.995 to 1), Dark, Alone (RMSEA = 0.059 to 0.106, SRMR = 0.006 to 0.021, CFI = 0.995 to 1), Dark, Others (RMSEA = 0.024 to 0.082, SRMR = 0.010 to 0.021, CFI = 0.996 to 1). All standardized factor loadings were large, significant, and similar across age groups. Internal consistency values were also strong across subscale and age group. At the subscale level, these subscales exhibited mild non-normality, but not overly-severe. Correlations among the four subscales were highly redundant across age groups (*r*s ranged from 0.72 to 0.91). These subscales were not, however, redundant with other study measures.

For the summated indexes in this variable domain, both the News-Related Avoidant Behaviors index and the Obligatory Behaviors index were severely non-normal, with positive skew, in the two older age groups. Severe non-normality was also evident for all four age groups for the Community Participation index. The Positive Avoidant Behaviors summated index and the No Behavioral Response summated index indicated reasonable normality and variation at the scale level. While correlations between these summated indexes were similar across age groups, some strong correlations were found between Positive Avoidant Behaviors and No Behavioral Response (*r*s ranged from 0.51 to 0.64) and Effect of Safety on Physical Activity measure and No Behavioral Response (*r*s = 0.63).

## 4 Discussion

The psychometric evaluation of the SAFE (sub)scales showed consistent factorial validity and internal consistency reliability across the majority of the measures, and importantly, across the four age groups. Therefore, the newly-developed and adapted single set of questions accurately measured target constructs for all groups, and the common measures can be used to facilitate analysis and interpretation of age-related patterns. This approach to simultaneous measurement development for the lifespan is the best way to determine whether common measures are feasible, and it is more time- and cost-efficient than a sequential development approach. Specifically, 14 of the 17 measures subjected to a test of factorial validity displayed statistically significant and strong factor loadings and internal consistency in the overall sample and across the age groups. The pattern of correlations for each (sub)scale with other (sub)scales and the 8 indexes did not exhibit any redundancy, with the exception of the Avoidant Behavior subscales. That is, each measure identified a degree of unique measurement variance in operationalizing a given construct.

Drilling down a bit more on this matter, establishing construct validity of empirical indicators has long been recognized (Cronbach, 1970) as an ongoing iterative process with the constant interplay between conceptualizing and empirical assessment. That said, following Messick’s (1995: 745) approach to unified construct validation, the current work has systematically assessed both the “structural aspect …the fidelity of the scoring structure to the structure of the construct domain at issue,” and the “generalizability aspect … [which] examines the extent to which score properties and interpretations generalize to and across population groups, settings, and tasks” (Messick, 1995: 745). The former was assessed by confirming at least roughly acceptable levels of interitem consistency for almost all of the indices. The latter was addressed by replicating roughly acceptable levels of interitem consistency across four different age groups of respondents. That said, we look forward to work by scholars, preferably with multiple methods, advancing work on the other relevant aspects of construct validation and particularly “the external aspects” gauging convergent and discriminant validities using multi trait – multimethod matrices (Campbell & Fiske, 1959), as well as tests of criterion validity with behavioral indicators. Such future studies with individual-level behavior-based criterion variables will further advance our understanding of potential differences in the construct validities of survey items that ask in different ways about walking, and walking and safety concerns. Putting aside disparities in other scientific quality benchmarks, until such evidence becomes available, disputes about how to word survey questions about walking and safety must necessarily remain un-resolved.

A further strength of the current work is that the avoidance and behavioral constraint items adopted here represent elaborations of items widely used as indicators for avoidance and behavioral constraint in the reactions to crime literature (Ferraro, 1994; Taylor, 2017). This further supports the “content aspect of construct validation” which “includes evidence of content relevance.”

While this psychometric evaluation was largely supportive of the validity and reliability of the SAFE measures, some items, and by implication measures, had weaknesses. The primary Macrolevel measure of neighborhood context, CPTED, could use improvement through additional item development. It should be emphasized that no existing scale or measures operationally defining the CPTED construct previously existed. The measures (four subscales) were developed to fully define physical design features (Taylor, 2017). The CPTED-Surveillance subscale in particular had lower factor loadings for two of the five items, and this was found across the four age groups. While the inclusion of these two items attenuated reliability to a degree, the overall reliability value is still fair given the number of items comprising the scale. Moreover, these two items are core concepts for CPTED construct. Given the conceptual strength of these items, and the minimal impact on reliability, these items can still be used but with great care. The Access Control and Territorial Reinforcement subscales, respectively, had limited psychometric evaluation. Given that these subscales were operationalized by only two items, factorial validity and internal consistency reliability could not be meaningfully assessed. The CPTED-Maintenance subscale, however, showed strong psychometric characteristics across the age groups.

Similar to the CPTED measure, the four Victimization subscales showed varying degrees of psychometric viability. All four subscales did exhibit strong factor validity. However, two of the four subscales, Recent and Past, respectively, exhibited a low degree of internal consistency. This was particularly pronounced for the Recent subscale. Not surprisingly, this/these measures also exhibited the most severe non-normality of all the measures. The low internal consistency is likely reflective of the low rates observed for some items from this/these specific subscales, and by implication the construct(s) being measured. For example, in the Adolescent age group, the item “In the past 12 months, how many times have you been the victim of property crimes [including theft, motor vehicle theft, burglary, vandalism]” from the Recent subscale did not significantly load on the factor in the CFA. This item is likely not as relevant for this younger age group as it is for the 3 older age groups. The low internal consistency values could also be a function of how Cronbach’s alpha is calculated. In addition to the inter-item correlations, Cronbach’s alpha is also a function of the number of items that compose a scale. In this case, there are 4 items per subscale. The magnitude of the standardized factor loadings, except for the Adolescent age group, all reflect fairly strong inter-item correlations. However, when the number of items is factored in to the calculation of alpha, the overall internal consistency value is necessarily attenuated. Given this, applied researchers should use these subscales with care.

The four Avoidant Behaviors subscales (Daylight, Alone; Daylight, Others; Dark, Alone; Dark, Others) all exhibited factorial validity and reliability across the age groups. However, the correlations among the four subscales indicated strong redundancy. For the majority of the correlations these values exceeded 0.70. This indicates a high degree of collinearity that could impact predictive models in which all subscales are added as simultaneous predictor variables. For this/these measures, participants’ responses did not reflect a difference between whether or not the target avoidant behavior was applicable to themselves/others or daylight/dark. Interestingly, these subscales were not redundant with other study measures, including related Avoidant measures from the same variable domain (e.g., Positive Avoidant Behaviors). At this point, it would not be prudent to use these four subscales in similar analyses. However, it does appear that the items from these four subscales could be aggregated for use as a General Avoidant scale.

This study is not without other general weaknesses. Not all of the SAFE measures could be validated using a factor-analytic approach. Many of the indexes were measured using a dichotomous rating scale. First, factor analysis is based on the assumption that items have an underlying continuum, even if measured in a binary format. This assumption could not be met with some of these indexes. Second, some of the indexes (e.g., Crime Resources Index) are more appropriately conceptualized and operationally defined using a checklist format, and thus are not represented meaningfully as a factor (or latent variable). Third, some measures were operationalized using as few as two items. These measures are similarly not amenable to factor-analytic evaluation to establish construct validity or reliability. However, these measures all showed significant unique measurement variance as reflected by the relatively small correlations found with the sub(scales) and other summated indexes. While these indexes were not amenable to formal factor-analytic procedures, their measurement form and properties suggest that they are reasonable measures to use in future studies. Finally, several sub(scales) and summated indices exhibited non-normality in the form of positive skewness at both the item- and scale level. This should not preclude the use of these measures in future studies. Measures of victimization, for example, are inherently skewed given their infrequent nature, but are vitally important in conceptual models of crime and physical activity (Patch et al., 2019).

## 5 Conclusion

These analyses supported the psychometric characteristics for the measures from SAFE, with the exceptions identified above. Importantly, the measurement properties were shown to be invariant (equal) across the four age groups of interest, thus facilitating cross-group comparisons in predictive models. This conclusion is consistent with the preliminary test–retest reliability evaluation reported in Patch et al. (2019). Although not reported due to space limitations, the findings reported herein were confirmed using additional statistical procedures specific to categorical data analysis (including item response theory) and accounting for clustering at the block group level. While we recommend these measures for use (with the caveat of not simultaneously among the Avoidant Behaviors measures), continued psychometric evaluation, and implementation of other forms of measurement invariance (Ployhart and Oswald, 2004), should be pursued to further refine these instruments and enhance their validity and reliability. The detailed evaluation of the measures, especially identification of weaknesses, will inform interpretation of results in subsequent papers.

## 6 Data availability statement

Data are housed at the University of California, San Diego. Data can be made available on request to Dr. James F. Sallis (jsallis@health.ucsd.edu).

## Funding

This study was funded by NHLBI grant #5R01HL117884.

## Declaration of Competing Interest

The authors declare that the research was conducted in the absence of any commercial or financial relationships that could be construed as a potential conflict of interest.
